# Retraction Note: Meta-analysis of contrast-enhanced ultrasound and contrast-enhanced harmonic endoscopic ultrasound for the diagnosis of gallbladder malignancy

**DOI:** 10.1186/s12911-022-01895-6

**Published:** 2022-06-07

**Authors:** Xue Liang, Xiang Jing

**Affiliations:** Department of Ultrasound, Tianjin Third Central Hospital, Tianjin Institute of Hepatobiliary Disease, Tianjin Key Laboratory of Artificial Cell, Artificial Cell Engineering Technology Research Center of Public Health Ministry, No. 83, Jintang Road, Hedong District, Tianjin, 300170 China

## Retraction note: BMC Medical Informatics and Decision Making (2020) 20:235 10.1186/s12911-020-01252-5

The authors have retracted this article. After publication it came to the authors attention that there were issues with the quality of the data used in this study. The authors, therefore, no longer have confidence in the results or conclusions of this study. All authors agree to this retraction.

